# Evidence for a Solid-Electrolyte Inductive Effect in the Superionic Conductor Li_10_Ge_1–*x*_Sn_*x*_P_2_S_12_

**DOI:** 10.1021/jacs.0c10735

**Published:** 2020-12-07

**Authors:** Sean P. Culver, Alexander G. Squires, Nicolò Minafra, Callum W. F. Armstrong, Thorben Krauskopf, Felix Böcher, Cheng Li, Benjamin J. Morgan, Wolfgang G. Zeier

**Affiliations:** † Institute of Physical Chemistry, Justus-Liebig-University Giessen, Heinrich-Buff-Ring 17, D-35392 Giessen, Germany; ‡ Center for Materials Research (LaMa), Justus-Liebig-University Giessen, Heinrich-Buff-Ring 16, D-35392 Giessen, Germany; § Department of Chemistry, University of Bath, Claverton Down, Bath BA2 7AY, United Kingdom; ∥ The Faraday Institution, Didcot OX11 0RA, United Kingdom; ⊥ Institute of Inorganic and Analytical Chemistry, University of Münster, Correnstrasse 30, 48149 Münster, Germany; # Jülich Centre for Neutron Science (JCNS), Forschungszentrum Jülich GmbH, Outstation at SNS, 1 Bethel Valley Road, Oak Ridge, Tennessee 37831-6473, United States

## Abstract

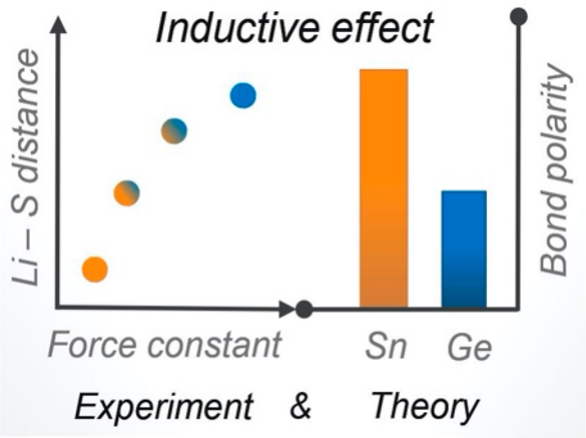

Strategies to enhance ionic conductivities in solid electrolytes typically focus on the effects of modifying their crystal structures or of tuning mobile-ion stoichiometries. A less-explored approach is to modulate the chemical bonding interactions within a material to promote fast lithium-ion diffusion. Recently, the idea of a solid-electrolyte inductive effect has been proposed, whereby changes in bonding within the solid-electrolyte host framework modify the potential energy landscape for the mobile ions, resulting in an enhanced ionic conductivity. Direct evidence for a solid-electrolyte inductive effect, however, is lacking—in part because of the challenge of quantifying changes in local bonding interactions within a solid-electrolyte host framework. Here, we consider the evidence for a solid-electrolyte inductive effect in the archetypal superionic lithium-ion conductor Li_10_Ge_1–*x*_Sn_*x*_P_2_S_12_. Substituting Ge for Sn weakens the {Ge,Sn}–S bonding interactions and increases the charge density associated with the S^2–^ ions. This charge redistribution modifies the Li^+^ substructure causing Li^+^ ions to bind more strongly to the host framework S^2–^ anions, which in turn modulates the Li^+^ ion potential energy surface, increasing local barriers for Li^+^ ion diffusion. Each of these effects is consistent with the predictions of the solid-electrolyte inductive effect model. Density functional theory calculations predict that this inductive effect occurs even in the absence of changes to the host framework geometry due to Ge → Sn substitution. These results provide direct evidence in support of a measurable solid–electrolyte inductive effect and demonstrate its application as a practical strategy for tuning ionic conductivities in superionic lithium-ion conductors.

## 1 Introduction

The past decade has seen numerous advances in the development and optimization of ionic conductors for solid-state battery applications,^1−3^ with particular attention directed toward lithium thiophosphates; these include the Li_6_PS_5_X argyrodites,^4−13^ the thio-LISICON phases,^14−18^ and Li_10_GeP_2_S_12_^19^ and its substitutional analogues.^20−25^ Within the Li_10_GeP_2_S_12_ family, room temperature ionic conductivities have been reported in excess of 10 mS cm^–1^,^19^ and similarly high ionic conductivities have been reported for other lithium thiophosphates.^11,12,19^ Understanding the factors that cause specific solid electrolytes to exhibit fast or slow ionic transport is a key research question, in part because such an understanding can inform the development of general “design rules” and support the identification and optimization of new fast-ion conducting materials,^26−29^ thereby broadening the pool of candidate solid electrolytes for future solid-state battery applications. A partial answer to the question of what makes some solid electrolytes much faster ionic conductors than others comes from an understanding of favorable structural motifs—for example, fast-ion conduction is favored in materials that possess highly connected networks of lithium diffusion pathways.^26^ Families of structurally related solid electrolytes, however, often exhibit room temperature ionic conductivities that vary by several orders of magnitude, highlighting the important role of chemical composition as a factor in understanding ionic conductivity trends between similar solid electrolytes.^30^

**Figure 1:**
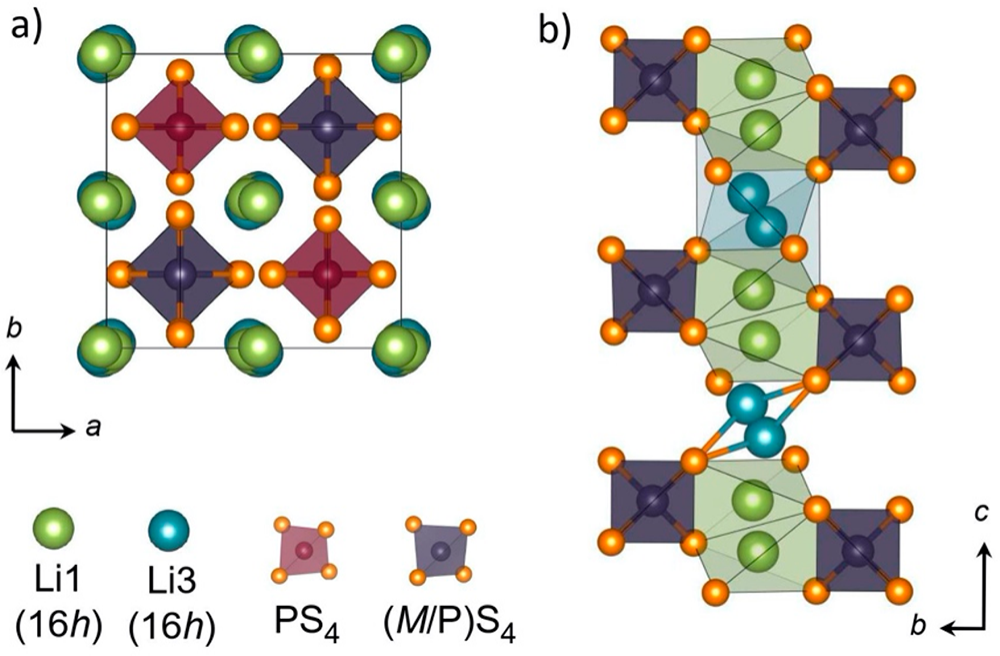
(a) View of the Li_10_MP_2_S_12_ unit cell, oriented along the *c*-axis, showing the one-dimensional primary lithium diffusion channels. (b) View of the same cell, oriented along the *a*-axis, showing the sequence of Li(1) and Li(3) lithium sites along the *c*-oriented diffusion channels and the positions of the adjacent (M/P)S_4_ tetrahedra.

The Li_10_MP_2_S_12_ (M = Si, Ge, Sn) thiophosphates adopt a tetragonal structure consisting of an anionic host framework of MS_4_^4–^ and PS_4_^3–^ tetrahedra that accommodates interstitial lithium ions (Figure 1). This host framework structure has open channels oriented along the [001] direction (Figure 1a) that enable fast lithium diffusion along *c*, while secondary conduction pathways between these *c*-oriented channels allow slower diffusion in the *a*–*b* plane.^31^ The dominant lithium diffusion process along *c* consists of lithium ions moving through alternating Li(1) and Li(3) sites (Figure 1b),^31,32^ where the rate of lithium diffusion depends on the underlying lithium-ion potential energy surface. The lithium-ion potential energy profile is determined both by the electrostatic interactions between lithium ions and by the interactions between the mobile lithium ions and the host framework.^31−34^ Chemical substitution may affect both the geometry and charge density distribution of the host framework, and both effects can modulate the lithium-ion potential energy profile, resulting in either increased or decreased lithium-ion conductivity.^21,35^

Chemical substitution within a solid-electrolyte host framework is a well-established strategy for enhancing the ionic conductivities of specific solid electrolytes.^26^ The selection of potentially beneficial framework atom substitutions is typically guided by extrapolating from trends observed in other solid-electrolyte families or by considering geometric models that aim to predict how particular substitutions might affect the structure of the host framework. One such model, for example, considers the increase in crystal volume that occurs when small framework atoms are replaced with larger substitute species. The resulting expansion of the electrolyte framework is expected to increase the interstitial volume available to diffusing lithium, thereby promoting lithium conduction.^36^ An alternative model considers how substitution of specific framework atoms can affect the local geometry of critical lithium diffusion pathways, in some cases causing an expansion or contraction of “bottlenecks”, thereby promoting or impeding lithium diffusion.^35^ Geometric models, such as these, often provide simple intuitive explanations for the conductivity trends observed within families of solid electrolytes. In some notable cases, however, observed conductivity trends run counter to those predicted on geometric grounds; chemical substitutions that would be expected to increase lithium-ion conductivities instead give the opposite effect and decrease ionic conductivities. One example of this contrary behavior is the isovalent substitution of Ge with the larger and more polarizable Sn within the Li_10_{Ge_,_Sn}P_2_S_12_ system, which was initially expected to produce an increased ionic conductivity because of an increase in overall lattice volume but in practice gives the opposite trend, with increasing Sn content giving a decreased lithium-ion conductivity.^23,35^

**Figure 2:**
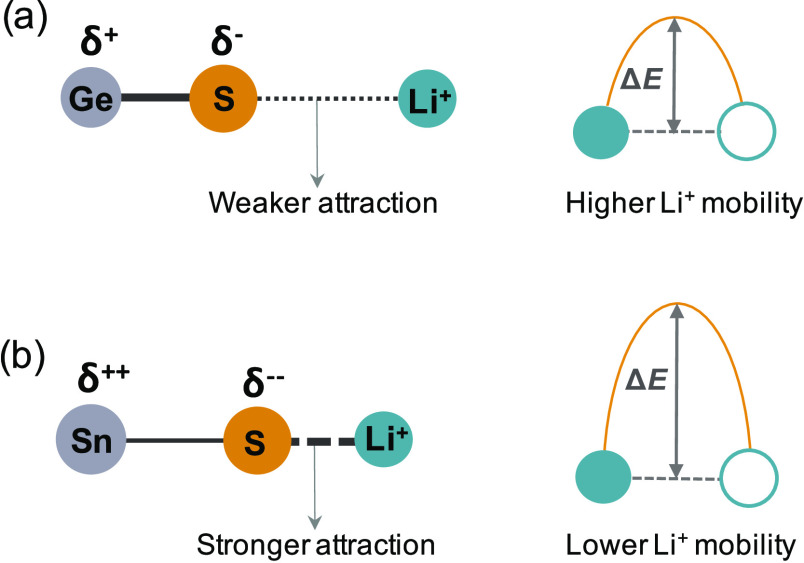
Schematic of the predicted difference in M–S···Li bonding for M = Ge vs M = Sn due to a hypothetical inductive effect. Changes in the M–S bonding modulate the Coulombic interaction between S and adjacent Li^+^ ions. In the LGPS structure, the Li(3) site is closer to the (M/P)S_4_ tetrahedra than the Li(1) site, which stabilizes Li occupation of the Li(3) site relative to occupation of the Li(1) site, producing an increased potential energy barrier for lithium diffusion.

Krauskopf et al.^35^ have recently suggested that this “inverted” response to chemical substitution might be explained by a solid-electrolyte inductive effect^35^—named by analogy to the well-known inductive effect model of Goodenough, which explains the effect of varying anion species on the intercalation voltages of lithium transition-metal compounds.^37−42^ In the case of Li_10_{Ge,Sn}P_2_S_12_, the solid-electrolyte inductive effect model predicts that the lower electronegativity of Sn vs Ge causes Sn–S bonds to be more polar than equivalent Ge–S bonds (see Figure 2),^a^ with Sn-bonded sulfur atoms therefore having an increased associated negative charge density than equivalent Ge-bonded sulfur atoms.^b^ This increased negative charge for Sn-bonded sulfur atoms compared to Ge-bonded sulfur atoms means that the electrostatic S···Li interaction between these sulfur atoms and nearby lithium ions is stronger in the Sn-substituted system than in the Ge analogue. In LGPS the Li(3) site sits closer to these {Ge,Sn}-bonded S atoms than the Li(1) site does; an increased S···Li interaction is therefore predicted to stabilize the Li(3) site compared to the Li(1) site. This increases the effective potential energy barrier for lithium diffusion along the Li(3)–Li(1) channels and results in a reduced lithium-ion conductivity.

Together with the proposal by Krauskopf et al.^35^ that an inductive effect might explain the observed lithium conductivity trend in Li_10_MP_2_S_12_, solid-electrolyte inductive effects have been invoked to explain anomalous conductivity trends in a number of other systems,^43^ including Na_11_Sn_2_PnS_12_ (Pn = P, Sb),^44^ Na_3_P_1–*x*_As_*x*_S_4_,^45^ Li_4–*x*_Sn_1–*x*_Sb_*x*_S_4_,^46^ and LiM_2_(PO_4_)_3_ (M = Zr, Sn).^47^ The solid-electrolyte inductive effect model is founded on simple chemical bonding concepts, making it an appealing model for explaining these unexpected conductivity trends. Yet there is no direct evidence that such an inductive effect does in fact exist. More specifically, it is not known to what extent varying the electronegativities of host framework atoms within a solid electrolyte can affect either the intraframework bonding interactions or the electrostatic interactions between the host framework and the mobile ions; nor is it clear whether such effects, if present, can modify ionic conductivities sufficiently to explain observed trends in solid-electrolyte conductivities.

Motivated by the question of whether the solid-electrolyte inductive effect does indeed exist, we have performed a combined experimental and computational study of the Li_10_Ge_1–*x*_Sn_*x*_P_2_S_12_ system. This study reveals a series of subtle structural and electronic changes produced by Ge^4+^ → Sn^4+^ substitution that are consistent with the model proposed by Krauskopf et al.^35^ Data from Raman spectroscopy and density functional theory (DFT) calculations show that the inclusion of lower electronegativity Sn produces weaker (more polar) M–S bonds within the MS_4_^4–^ tetrahedra, and Rietveld refinements against high-resolution temperature-dependent neutron diffraction data show that this decrease in M–S bond strength is correlated with shorter S^2–^–Li^+^ distances and increased Li^+^ site occupation around the S^2–^ ions, which suggests a stronger S^2–^···Li^+^ interaction in the Sn-substituted system. DFT-calculated lithium binding energies provide further complementary evidence that Sn substitution increases the strength of the S^2–^···Li^+^ interactions. Our DFT calculations also show that these changes in M–S bonding and S^2–^···Li^+^ interactions are coupled to a modulation of the Li^+^ potential energy profile along the Li(3)–Li(1) diffusion pathway in the channels: substituting Ge^4+^ for Sn^4+^ gives a higher potential energy maximum for single-lithium-ion motion. Further calculations for the Sn-substituted system, but with a fixed geometry corresponding to the Ge-substituted analogue, show that Sn substitution increases the height of the Li^+^ diffusion profile even in the absence of changes in host framework geometry, providing further evidence for an electronic inductive effect.

When considered together, these results provide compelling evidence in support of a solid-electrolyte inductive effect in the Li_10_Ge_1–*x*_Sn_*x*_P_2_S_12_ system. These data also illustrate how information about subtle changes in host framework bonding and framework mobile ion interactions arising from framework atom substitution can be obtained through a combination of experimental and computational techniques and how this can provide a clearer understanding of the chemical effects responsible for modulating ionic conductivities in families of structurally similar lithium-ion solid electrolytes.

## 2 Experimental Methods

### Synthesis

All preparations and sample treatments were performed under an argon atmosphere (O_2_ < 1 ppm, H_2_O < 5 ppm). Li_10_Ge_1–*x*_Sn_*x*_P_2_S_12_ was synthesized using the following procedure: the starting materials of lithium sulfide (Li_2_S, Sigma-Aldrich, 99.98%), phosphorus pentasulfide (P_2_S_5_, Sigma-Aldrich, 99%), elemental sulfur (S_8_, Arcos Organics, >99.999%), germanium sulfide (GeS, Sigma-Aldrich, 99.99%), and tin sulfide (SnS, Sigma-Aldrich, >99.99%) were mixed in the appropriate stoichiometric ratio. Additionally, a 3 wt % excess of sulfur was added to the mixture to compensate for sulfur loss at higher temperatures. The resulting mixture (3 g) was ball-milled (Fritsch Pulverisette 7 premium line) at 400 rpm using a ZrO_2_ milling set (80 mL bowl with 90 g of 3 mm diameter milling media). The milling was performed for 48 h with intermediate cooling times (i.e., 15 min of cooling after every 10 min of milling) to prevent excessive heating of the samples. Twice during this process, the grinding bowl was opened, and the resultant mixture was ground to obtain a uniform precursor. The resultant precursor (1 g) was pressed into pellets, which were then sealed under vacuum into 10 mm inner diameter quartz ampules. The ampules were heated in a tube furnace to 773 K (at 27 K h^–1^), annealed for 20 h, and then cooled to room temperature. To reduce the formation of side phases in Li_10_SnP_2_S_12_, this pellet was reheated at 873 K for 48 h (at 50 K h^–1^).

### Neutron Powder Diffraction

Neutron powder diffraction data were collected using the Spallation Neutron Source (SNS) POWGEN diffractometer at Oak Ridge National Laboratory.^48^ Approximately 3 g of sample was loaded into an 8 mm diameter cylindrical vanadium sample can. Using a center wavelength of 0.8 Å with a *d*-spacing from 0.2 to 6.0 Å, we collected data for ∼3 h in the high-resolution mode at each temperature.

### Rietveld Analysis

Rietveld refinements were performed using the TOPAS-Academic V6 software package,^49^ using a convolution of back-to-back exponential and the Thompson–Cox–Hastings pseudo-Voigt function for the profiles. The following parameters were initially refined: (1) scale factor, (2) background coefficients, (3) peak shape, (4) lattice constants, (5) fractional atomic coordinates, and (6) isotropic atomic displacement parameters. Additionally, Sn was allowed to occupy the Wyckoff 4d positions and constrained to the value of *g*(Ge) + *g*(Sn) = 0.5. Bond lengths and polyhedral volumes were extracted from the Vesta software package (ver. 3).^50^

### Raman Analysis

Raman spectra were collected using a Senterra Raman spectrometer (Bruker) with an excitation wavelength of 532 nm. Data collection was performed in a spectral range from 55 to 1555 cm^–1^ by using a 20× objective and a power of 0.2 mW. The powder samples were placed on glass substrates inside a glovebox, framed with silicone vacuum grease, and sealed airtight with a cover glass to ensure an inert atmosphere during measurement.

### Computational Methods

All density functional theory (DFT) calculations were performed using VASP^51−53^ with valence electrons described by a plane-wave basis. Interactions between core and valence electrons were described by using the Projector Augmented Wave (PAW) method with valence electron configurations of Li [2s^1^], Ge [4s^2^ 4p^2^], Sn [5s^2^ 5p^2^], P [3s^2^ 3p^3^], and S [3s^2^ 3p^4^].^54^ All calculations used the Generalized Gradient Approximation (GGA) functional PBEsol.^55^ Calculations with a fixed cell volume used a plane-wave cutoff of 500 eV, while calculations with a variable cell volume used an increased cutoff of 650 eV to minimize errors due to Pulay stress. Geometry optimizations were deemed converged when all atomic forces were smaller than 0.01 eV Å^–1^. All calculations were spin-polarized and used a Monkhorst–Pack grid for sampling **k**-space, with the minimum spacing between ***k***-points set to 0.3 Å^–1^.

To quantify charge distributions and bonding characters from our DFT calculations, we assign net atomic charges, calculated by using the DDEC6 methodology^56^ as implemented in the CHARGEMOL package,^57^ and integrated Crystal-Orbital Hamilton Populations (iCOHP), calculated by using the LOBSTER code.^58−60^ Within LOBSTER, the *vaspfitpbe2015* basis functions were used to map the VASP plane-wave basis set onto local orbitals. To sample single-lithium-ion diffusion potential energy profiles along the *c* channels, we performed climbing-image nudged–elastic-band (c-NEB)^61^ calculations, using the *pathfinder* algorithm of Rong et al.^62^ to obtain an initial approximation of each minimum-energy-barrier path. To estimate the effect of chemical substitution on lithium transport in the absence of any structural change, the energy of each structure along the c-NEB pathway for both Li_10_GeP_2_S_12_ and Li_10_SnP_2_S_12_ was then recalculated, substituting Ge for Sn, and vice versa, fixing all cell parameters and ionic positions.

To estimate changes in the S–Li interaction for M = Sn and Ge, we calculated “vertical” (unrelaxed) Li^+^ vacancy formation energies () for all Li atoms as a function of nearest-neighbor S–Li distance, with reference to the S atoms that constitute the vertices of the SnS_4_^4–^ tetrahedra. The Li^+^ vacancy formation energies, , were calculated as
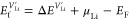
1where  is given by the difference in energy between a stoichiometric defect-free supercell and an equivalent cell containing a single Li^+^ vacancy, with all other atoms held fixed in place. μ_Li_ is the chemical potential of the Li atom to be removed from the cell, and *E*_F_ is the chemical potential of the electron to be added (Fermi energy), referenced to the valence band maximum, with the bulk electrostatic potentials of the defect and pristine cell aligned.^63^

Calculations of formation energies of charged defects using periodic models, as for the Li^+^ vacancy considered here, typically include an image-charge energy term (*E*_icc_) to correct for a shift in total energy due to the artificial interaction of a defect with its periodic images. This correction requires calculation of the dielectric tensor, which is ill-defined within a DFT framework for an intrinsically disordered system such as LGPS. For the results presented here, we do not include an explicit image-charge correction and instead attempt to minimize the variation in the neglected correction term—which scales approximately as *L*^–3^, where *L* is the length of the simulation cell^64^—by performing our defect calculations in large 400-atom 2 × 2 × 2 Li_10_GeP_2_S_12_ supercells. We have estimated the magnitude of the (neglected) image-charge correction term by performing an explicit calculation for a *V*_Li_^′^ vacancy in a pseudo-ordered structure of Li_10_GeP_2_S_12_ taken from the Materials Project,^65^ using the approach of Lany and Zunger,^63^ which gives a representative value of *E*_icc_ = 0.05 eV.

Because Li_10_GeP_2_S_12_ is intrinsically lithium disordered, the S–Li interaction may not be well characterized by considering a single lithium-vacancy formation energy. To account for this lithium disorder, we have sampled the distribution of vacancy formation energies as a function of S–Li distance from a set of 160 lithium configurations. These representative 160 configurations were selected from an initial set of 500000 structures, with candidate structures selected by ranking their approximate electrostatic energies, by using the Ewald summation functionality in the PYMATGEN package.^66^

A data set containing DFT calculation inputs and outputs is available at the University of Bath Data Archive, published under the CC-BY-4.0 license.^67^ The data set also includes analysis scripts, published under the MIT license, used to postprocess the DFT data and to plot Figures 4 and 6. The data analysis scripts use the Python packages PYMATGEN,^66^ NUMPY,^68^ PANDAS,^69^ and MATPLOTLIB.^70^

## 3 Results

### General Structural Characterization

To characterize the influence of Ge^4+^ → Sn^4+^ substitution in the Li_10_Ge_1–*x*_Sn_*x*_P_2_S_12_ series, compounds were synthesized with four varying stoichiometries—nominally *x* = 0, 0.33, 0.67, and 1. Because of the low X-ray form factor of Li^+^, high-resolution neutron diffraction data were collected in the temperature range 300–500 K to allow the subtle effects of Sn substitution on the lithium substructure to be resolved. Representative Rietveld refinements of the room-temperature neutron diffraction data for the Li_10_Ge_1–*x*_Sn_*x*_P_2_S_12_ compounds are shown in Figure S1. All constraints used in the refinements are tabulated in Table S1, and the resulting structures at all temperatures are included as crystallographic information format (CIF) files in the Supporting Information. The reflections within the isostructural Li_10_Ge_1–*x*_Sn_*x*_P_2_S_12_ patterns were indexed to the tetragonal Li_10_Ge_1–*x*_Sn_*x*_P_2_S_12_ structure, crystallizing in the *P*4_2_/*nmc* space group. The room temperature structural data (Figure S2a) show that substitution of the larger Sn^4+^ cation (0.55 Å) for Ge^4+^ (0.39 Å) within a tetrahedral coordination environment^71^ causes linear increases in the lattice parameters *a* and *c*, in the *c*/*a* ratio of the tetragonal unit cell, and in the unit cell volume. Thus, Vegard’s law is obeyed, confirming the successful synthesis of homogeneous solid solutions. With increasing temperature, the lattice parameters and unit-cell volumes increase linearly, while the *c*/*a* ratios decrease (Figure S2b), in good agreement with literature data.^23^ The effect of temperature and composition on all polyhedral volumes is included in the Supporting Information (Figure S3). The (M/P)S_4_ tetrahedral volume increases with substitution, whereas the PS_4_ tetrahedral volume remains constant, which agrees with literature data.^35^ Interestingly, whereas Li(1), Li(2), and Li(3) polyhedral volumes increase with Sn substitution, the rather immobile Li(4) site exhibits no significant change upon Sn introduction.

### Bond Strength Indicators and Changing M–S Bonding Interactions

**Figure 3:**
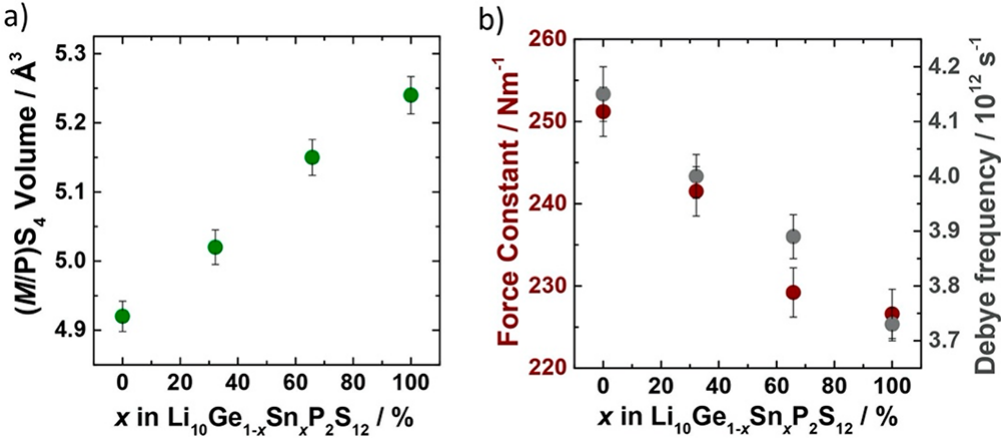
Bond strength indicators in Li_10_Ge_1–*x*_Sn_*x*_P_2_S_12_. (a) Temperature-averaged (M/P)S_4_ polyhedral volumes and (b) weighted averaged force constants of the MS_4_ tetrahedra (dark red) and the Debye frequencies (literature values from ref (35), dark gray) for Li_10_Ge_1–*x*_Sn_*x*_P_2_S_12_ are also provided.

The linear increase in Li_10_Ge_1–*x*_Sn_*x*_P_2_S_12_ unit-cell volume with increasing Sn content is mirrored by an increased (M/P)S_4_ tetrahedral volume and an increased M–S bond distance (Figure 3a). This increased bond distance suggests that Ge^4+^ → Sn^4+^ substitution causes a decrease in M–S bond strength. The Raman spectra for the Li_10_Ge_1–*x*_Sn_*x*_P_2_S_12_ series (Figure S4) support this interpretation; while the Raman shift for the symmetric stretching mode of the PS_4_^3–^ units remains unchanged throughout the series, the analogous vibrational mode of the SnS_4_^4–^ units, ν_1_(A_1_), is consistently observed at lower wavenumbers relative to the GeS_4_^4–^ units. This behavior can be attributed both to the changes in bond lengths and to the difference in electronegativity between Ge and Sn. Considering the M–S bond in the MS_4_^4–^ tetrahedra as a harmonic spring, the force constant *K* can be related to the Raman shift via^72^
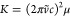
2where *ṽ* is the Raman shift and μ is the reduced mass. Because the central atoms *M* are at rest during the symmetric A_1_ stretches of the GeS_4_^4–^ and SnS_4_^4–^ tetrahedra, μ can be replaced by the mass of S,^72^ which allows the observed Raman shifts across the Li_10_Ge_1–*x*_Sn_*x*_P_2_S_12_ series to be expressed as force constants for the A_1_ vibration modes. To account for changes in the Ge/Sn ratios, and thus for changes in the relative contributions to each vibration, we have reweighted these force constants based on the Rietveld-refined compositions to obtain an averaged descriptor. Increasing Sn content is associated with a decrease in the weighted force constant (Figure 3b), which is consistent with a corresponding decrease in average M–S bond strength. As a complementary measure, we also consider the Debye frequencies for the Li_10_Ge_1–*x*_Sn_*x*_P_2_S_12_ series (Figure 3b), which describe an “average bond strength” for each composition.^35^ The decrease in the Debye frequency with increasing Sn content is consistent with the trend observed for the Raman-spectra-derived force constants and further supports the proposition that Ge → Sn substitution decreases the strength of M–S bonds in Li_10_Ge_1–*x*_Sn_*x*_P_2_S_12_.

**Figure 4:**
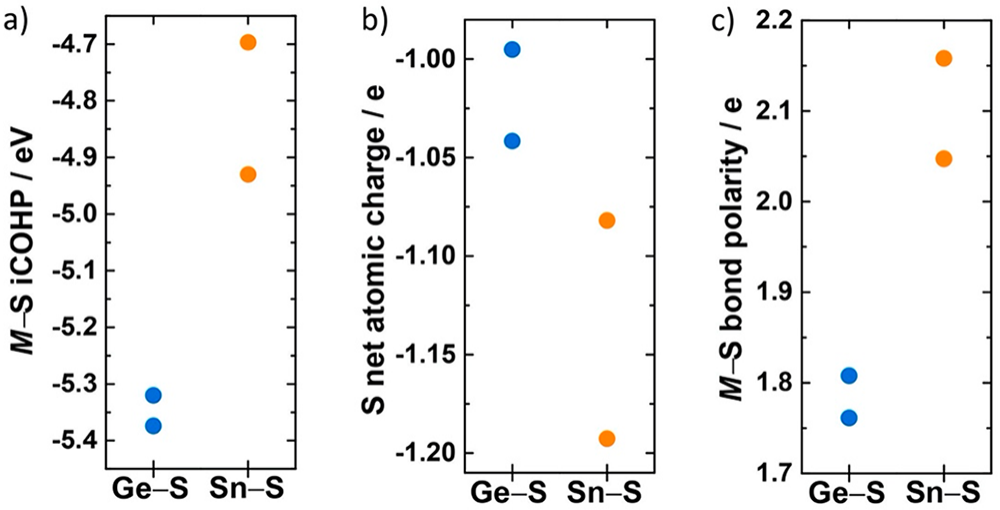
(a) M–S integrated crystal-orbital Hamilton populations (iCOHP) calculated for Li_10_MP_2_S_12_ (M = Ge, Sn), (b) sulfur net atomic charges (NAC), and (c) M–S bond polarities. Consistently higher net atomic charges on sulfur can be found for M = Sn, which is correlated with a weaker bonding interaction between Sn and S relative to Ge and S.

To explore how the choice of M = {Ge, Sn} affects the M–S bonding character, we have calculated integrated crystal-orbital Hamilton populations (iCOHPs) for the M–S bonds in Li_10_Ge_0.5_Sn_0.5_P_2_S_12_. These iCOHP values are plotted in Figure 4a and are indicative of bonding strength within the respective MS_4_ tetrahedra: more negative values indicate stronger and more covalent bonding, while more positive values indicate weaker and more polar bonding.^58−60^ The more negative values obtained for the GeS_4_ tetrahedra compared to the SnS_4_ tetrahedra suggest stronger bonding interactions for these Ge–S bonds compared to the equivalent Sn–S bonds, which agrees with the trends observed for the M–S force constants and Debye frequencies.

To investigate whether these changes in bond strength are coupled to a measurable change in the charge density associated with the M-bonded S^2–^ ions, we have also calculated net atomic charges for the Li_10_GeP_2_S_12_ and Li_10_SnP_2_S_12_ end members (Figure 4b). This analysis assigns larger more negative charges to Sn-bonded S^2–^ ions in Li_10_SnP_2_S_12_ than to equivalent Ge-bonded S^2–^ ions in Li_10_GeP_2_S_12_. To quantify the M–S bond polarity in Li_10_GeP_2_S_12_ and Li_10_SnP_2_S_12_, we computed the differences in net atomic charge values between the Ge or Sn ions and their coordinating S^2–^ ions. We again find a subtle but clear difference in bonding character between these two end members, with the Ge–S bonds in Li_10_GeP_2_S_12_ being less polar in character than the Sn–S bonds in Li_10_SnP_2_S_12_.

These bond strength indicators (bond stretch force constants and iCOHP values) and bond polarity data (net atomic charges) together give a coherent picture of how substituting Sn for Ge in Li_10_MP_2_S_12_ affects the M–S bonding. Ge–S bonding is stronger and less polar than Sn–S bonding, and Ge-bonded S atoms in Li_10_GeP_2_S_12_ have smaller (less negative) associated charges than Sn-bonded S atoms in Li_10_SnP_2_S_12_. Each of these observations is consistent with the predictions of the inductive effect model.

### Modulating S–Li Interactions and the Effect on the Li^+^ Substructure

Our DFT calculations, discussed above, predict that S(2) atoms carry more negative charge when bonded to Sn than to Ge. This increased negative charge is expected to correspond to a stronger Coulombic interaction between these S(2) atoms and nearby lithium ions, which may cause a change in the lithium-ion substructure. The *c*-oriented lithium diffusion pathway consists of alternating Li(1) and Li(3) sites, with the Li(3) sites closest to the S(2) framework atoms. We would therefore expect that a change in the S(2)···Li interaction with increasing Sn content would most strongly affect lithium occupying the Li(3) sites compared to lithium occupying the more distant Li(1) sites.

**Figure 5:**
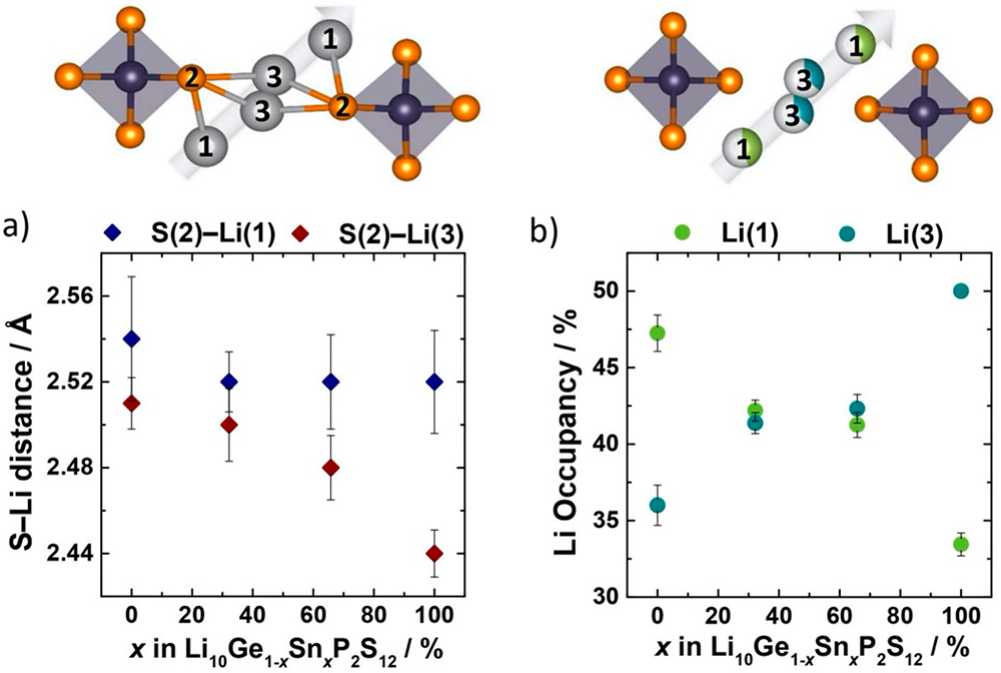
Variation in lithium substructure with Sn content in Li_10_Ge_1–*x*_Sn_*x*_P_2_S_12_: (a) the S(2)–Li(1) and S(2)–Li(3) distances and (b) Li(1) and Li(3) occupancies along the main diffusion channel. All shown data obtained from the refinements at 300, 375, 450, and 500 K have been averaged for visual clarity and to highlight the persistence of the trends within the studied temperature range. Error bars show the standard deviations for these data when combining all temperatures.

Figures 5a and 5b show plots of the S(2)–Li(1) and S(2)–Li(3) distances and the Li(1) and Li(3) percentile occupancies as functions of *x*(Sn), as obtained from Rietveld refinements against neutron diffraction data. These plots show temperature-averaged data to highlight the persistence of the observed trends across the studied temperature range. The full data set containing values obtained at each temperature is included in the Supporting Information (Figure S5).

As the Sn content increases, the S(2)–Li(3) distance decreases, while the S(2)–Li(1) distance is largely unchanged (Figure 5a). This is consistent with the expectation that changes in the S(2) charge density affect neighboring Li(3) lithium ions more strongly than more distant Li(1) lithium ions. Increasing the degree of Sn substitution also produces an increase in the Li(3) site occupancy and a corresponding decrease in the Li(1) site occupancy (Figure 5b). This redistribution of lithium ions from Li(1) to Li(3) sites is also consistent with a picture of lithium ions being more strongly attracted to Li(3) sites vs Li(1) sites as Ge is progressively substituted by Sn.

### Ge → Sn Substitution Effects on the Li^+^ Ion Potential Energy Surface

**Figure 6:**
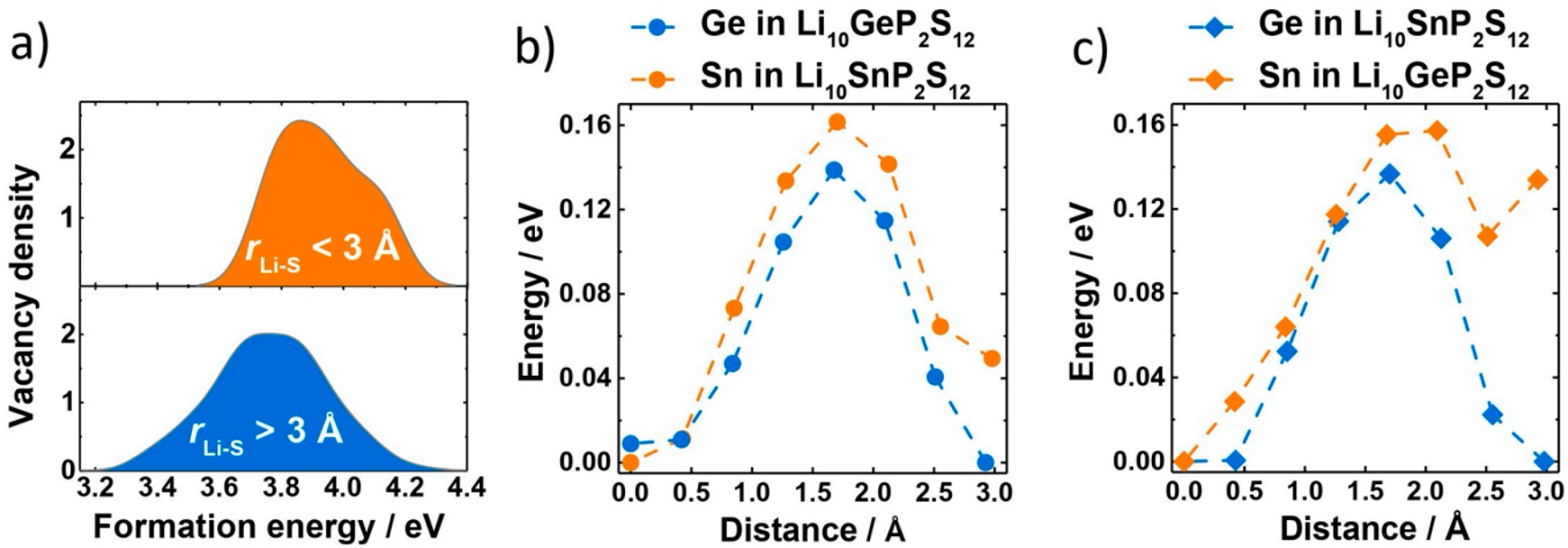
(a) Computed probability distribution of Li vacancy formation energies in a Li_10_GeP_2_S_12_ supercell containing one single Sn ion. The blue distribution shows vacancies far (≥3 Å) from the sulfur ions bonded to Sn; the orange shows those vacancies near the same S^2–^ ions with a distance <3 Å. The distribution suggests that Li^+^ is more strongly bound to SnS_4_^4–^ tetrahedra compared to GeS_4_^4–^. This evidence is confirmed by nudged elastic band calculations performed for (b) the Li_10_GeP_2_S_12_ and Li_10_SnP_2_S_12_ structures first as well as in (c) the respective structure after switching of Ge with Sn, and vice versa, keeping all structural parameters fixed. Consistently higher activation barriers are found for Sn, irrespective of the starting structure, showing an influence of the charge density itself.

To corroborate the stabilizing effect of Sn substitution on Li^+^ ions occupying nearby Li(3) sites, we performed a further series of DFT calculations in which we computed the Li^+^ vacancy formation energies () for a set of Li_10_GeP_2_S_12_ supercells each containing one Sn ion. These Li^+^ vacancy formation energies give a relative measure of the “binding energy” of Li^+^ at different positions within each supercell; a larger vacancy formation energy corresponds to a more stable Li^+^ position. Figure 6a shows the resulting distributions of calculated Li^+^ vacancy formation energies, classified according to whether the Li^+^ ion removed is originally located less than 3 Å of a Sn-bonded S(2) atom or not. The vacancy formation energies for Li^+^ ions close to Sn-bonded S(2) atoms are shifted to higher energies relative to the vacancy formation energies for Li^+^ ions that sit further away; that is, there is a greater energy cost to remove lithium ions from Sn–S(2) adjacent positions. This agrees with the interpretation of our neutron diffraction refinement data that Li^+^ ions are indeed more strongly bound to S in SnS_4_^4–^ tetrahedra than to S in GeS_4_^4–^ tetrahedra.

Because the primary diffusion channels in Li_10_Ge_1–*x*_Sn_*x*_P_2_S_12_ are composed of alternating Li(3) and Li(1) sites, the enhanced binding of Li^+^ ions at Li(3) sites vs Li(1) sites with increasing Sn content is expected to correspond to a modulation of the potential energy profile for Li^+^ ions moving within these *c*-oriented diffusion channels. To better quantify the effect of Ge → Sn substitution on the Li^+^ ion potential energy profile along the Li(3)–Li(1) diffusion channels, we consider potential energy profiles obtained from a series climbing-image nudged elastic band (c-NEB) calculations for a single Li^+^ ion moving from the Li(3) site to the Li(1) site. Li diffusion in Li_10_GeP_2_S_12_ proceeds by the concerted stringlike motion of groups of lithium ions,^73^ and NEB pathways for individual lithium ions therefore should not be equated with the true microscopic free energy barrier for lithium motion (which determines the activation energy for Li^+^ conduction). In this case, however, we are interested in local differences in the potential energy surfaces as a function of Ge → Sn substitution, and we consider these single-Li^+^ NEB barriers as a proxy metric for the “roughness” of the true many-body potential energy surface. The c-NEB profiles for Li^+^ diffusion in Li_10_GeP_2_S_12_ and in Li_10_SnP_2_S_12_ are shown in Figure 6b. These profiles were computed following the standard c-NEB procedure, allowing all images along the diffusion path to fully relax within the c-NEB constraints. These “relaxed” c-NEB profiles show a larger potential energy barrier for Li(3) → Li(1) Li movement in Li_10_SnP_2_S_12_ than in Li_10_GeP_2_S_12_, in agreement with conductivity trends from experiment and diffusion coefficients from previous molecular dynamics simulations of Li_10_(GeSn)P_2_S_12_.^21^ This result, again, agrees with the qualitative predictions of the solid-electrolyte inductive effect model.

### Decoupling Geometric and Electronic Effects of Ge → Sn Substitution

The substitution of Ge for Sn in Li_10_GeP_2_S_12_ does not only affect the chemical bonding and charge distribution within the host framework; it also changes the host framework geometry. It is therefore possible that even though our data provide strong evidence for a solid–electrolyte inductive effect in Li_10_Ge_1–*x*_Sn_*x*_P_2_S_12_, this might not be the cause of the conductivity trend observed in experiment—instead, the observed effect may be due to the geometric effects of Ge → Sn substitution.^35^ To resolve the electronic and geometric contributions to the potential energy barrier difference predicted for our fully relaxed c-NEB calculations, we performed a second set of calculations with Sn fully substituted into Li_10_GeP_2_S_12_ and Ge fully substituted into Li_10_SnP_2_S_12_, with each image along the diffusion pathway held fixed at the original geometry. In other words, we compute an approximate barrier for Li_10_SnP_2_S_12_ fixed at the optimized Li_10_GeP_2_S_12_ geometries and for Li_10_GeP_2_S_12_ fixed at the optimized Li_10_SnP_2_S_12_ geometries. If the relative potential energy barriers for Li_10_GeP_2_S_12_ and for Li_10_SnP_2_S_12_ depend only on the difference in host framework geometry produced by Ge → Sn substitution, we would expect the relative barriers from these cation-exchanged fixed-geometry calculations to give a lower barrier for Li_10_SnP_2_S_12_ (computed by using the optimized Li_10_GeP_2_S_12_ geometries) and a higher barrier for Li_10_GeP_2_S_12_ (computed by using the optimized Li_10_SnP_2_S_12_ geometries). Instead, we see the opposite trend (Figure 6c). The approximate potential energy barrier is higher for Li_10_SnP_2_S_12_ even when the geometry of the diffusion pathway is that of fully relaxed Li_10_GeP_2_S_12_. Providing these Li^+^ ion potential energy barriers are effective descriptors of the variation in the true many-body free energy surface in Li_10_Ge_1–*x*_Sn_*x*_P_2_S_12_, this result suggests that the observed conductivity trend cannot be attributed solely to geometric effects and that electronic effects, such as those described by the solid-electrolyte inductive effect model, have an experimentally significant effect on the ionic conductivities of the Li_10_Ge_1–*x*_Sn_*x*_P_2_S_12_ series.

### Structure–Transport Correlations

**Figure 7:**
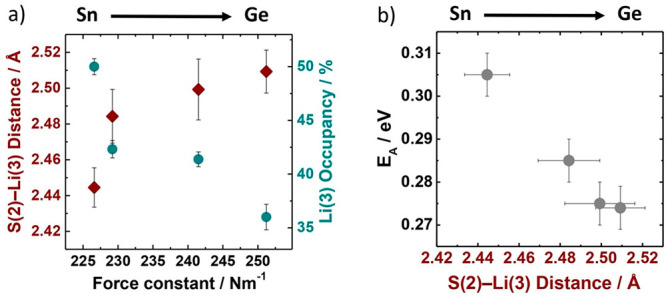
(a) Changes in the S(2)–Li(3) distances and Li(3) occupancies with changing weighted force constant in Li_10_Ge_1–*x*_Sn_*x*_P_2_S_12_. (b) S(2)–Li(3) distance vs previously reported Li^+^ ion conductivity activation energies. Activation energies are digitized from ref (35) and reported in Table S2.

While the NEB analysis above indicates that the electronic effects of Ge → Sn substitution can produce a meaningful change in the lithium-ion potential energy surface even in the absence of competing geometric effects, this does not mean that geometric effects play no role in the observed conductivity trend in Li_10_Ge_1–*x*_Sn_*x*_P_2_S_12_ nor that there is not a geometric component to the solid-electrolyte inductive effect. Ge → Sn substitution causes the weighted force constants of the MS_4_^4–^ polyhedra to decrease, which is correlated to decreased S(2)–Li(3) distances and increased Li(3) occupancies (Figure 7a). As discussed above, we attribute this response of the lithium substructure to the greater electron density on Sn-bonded S(2) atoms compared to Ge-bonded S(2) atoms, which is a consequence of the weaker Sn–S(2) bonding vs Ge–S(2) bonding. We also note a strong correlation between the S(2)–Li(3) distance and experimentally reported activation energies for lithium conduction (Figure 7b); with increasing Sn content and decreasing S(2)–Li(3) distances the ionic conductivity activation energy increases significantly, which we attribute to the increased strength of the S(2)···Li(3) interaction in Sn-substituted Li_10_Ge_1–*x*_Sn_*x*_P_2_S_12_.

## 4 Summary and Conclusion

The solid-electrolyte inductive effect model offers a possible explanation for the otherwise anomalous conductivity trend observed for Li_10_Ge_1–*x*_Sn_*x*_P_2_S_12_ as well as for a number of other solid electrolyte families.^43−45,47^ This model proposes that in Li_10_Ge_1–*x*_Sn_*x*_P_2_S_12_ the lower electronegativity of Sn compared to Ge causes Sn–S bonds to be weaker and more polar than analogous Ge–S bonds. The increased polarity of these Sn–S bonds corresponds to a larger (more negative) charge density associated with the Sn-bonded S atoms, which in turn causes a stronger Coulombic attraction between these S atoms and nearby Li^+^ cations. Li^+^ ions adjacent to Sn-bonded S atoms are therefore expected to be more “tightly bound”—that is, they have lower potential energies—relative to Li^+^ ions further away, than otherwise equivalent Li^+^ ions adjacent to Ge-bonded S atoms. This change in S···Li interaction strength is then predicted to change the profile of the potential energy surface for lithium diffusion along the *c*-oriented one-dimensional channels, giving a higher barrier to diffusion in Li_10_SnP_2_S_12_ than in Li_10_GeP_2_S_12_, thereby explaining the reduced room temperature ionic conductivity and higher lithium conduction activation energy observed in experiments.^43−45,47^

While this solid–electrolyte inductive effect model is chemically intuitive, and potentially explains a number of otherwise anomalous conductivity trends, there has previously been insufficient data to confirm whether this mechanism does indeed produce a significant effect in lithium-ion solid electrolytes, including Li_10_Ge_1–*x*_Sn_*x*_P_2_S_12_. To address this issue, we have conducted a combined high-resolution temperature-dependent neutron diffraction, Raman spectroscopy, and DFT study of the variation in lithium substructure, bonding interaction, and lithium-ion potential energy profile in the Li_10_Ge_1–*x*_Sn_*x*_P_2_S_12_ series. Our combined experimental and computational results provide direct evidence for a solid-electrolyte inductive effect in this family of superionic solid electrolytes. Our observed variations in M–S distances, force constants from Raman data, Debye frequencies, and DFT data show that substituting Sn into Li_10_Ge_1–*x*_Sn_*x*_P_2_S_12_ does indeed produce a decrease in M–S bonding strength, leading to an increasing electron density on S. Further analysis of S–Li distances and Li site occupancies alongside DFT-calculated binding energies corroborates a stronger Coulombic attraction between Li^+^ and S^2–^. Additional c-NEB DFT calculations indicate that these changes in M–S and S···Li interactions are associated with an increased potential energy barrier for Li diffusing along the *c*-oriented diffusion channels. These data are all consistent with the predictions of the solid-electrolyte inductive effect model^35^ and provide strong supporting evidence for the existence of this inductive effect in the Li_10_Ge_1–*x*_Sn_*x*_P_2_S_12_ family of superionic solid electrolytes. Finally, analysis of the potential energy profile along the *c*-oriented diffusion channels for Li_10_SnP_2_S_12_ fixed at Li_10_GeP_2_S_12_ geometries and for Li_10_GeP_2_S_12_ fixed at Li_10_SnP_2_S_12_ geometries shows that the predictions of the solid-electrolyte inductive effect model hold even in the absence of the structural changes that accompany Sn substitution in real materials, suggesting that the inductive effect produces a sufficiently large perturbation to the lithium-ion potential energy profile to be experimentally meaningful, even when decoupled from structural changes to the host framework.

While the data presented here provide evidence for an experimentally significant solid-electrolyte inductive effect in the Li_10_Ge_1–*x*_Sn_*x*_P_2_S_12_ system, it is unknown to what extent analogous inductive effects may be a factor in the relative ionic conductivities of other families of solid electrolytes.^43−45,47^ The Li_10_Ge_1–*x*_Sn_*x*_P_2_S_12_ system may be an exceptional case because of the particular geometry of the host framework—in this crystal structure the M-bonded S anions, i.e., those directly affected by Ge → Sn substitution, are arranged along the sides of the main *c*-oriented conduction pathways and may therefore exhibit a particularly strong influence on Li^+^ ion diffusion. To what extent the inductive effect does, or does not, play a role in controlling ionic transport in other families of solid electrolytes therefore remains an intriguing question for future study.

## Supporting Information Available

Representative Rietveld refinements against time-of-flight neutron diffraction data and the used constraints; all the experimental Raman spectra and the shifts relative to the vibration modes; literature values of activation energy and ionic conductivity of Li_10_Ge_1–*x*_Sn_*x*_P_2_S_12_ (PDF)
Crystallographic information format (CIF) files and all structural data as obtained from the refinements for all explored temperatures and compositions (ZIP)

## Footnotes

a The electronegativities of Ge^4+^ and Sn^4+^ are 2.0 vs 1.7 Pauling units on the Allred–Rochow scale of electronegativity with 1 Pauling unit = 6 eV·e^–174^ and 11.1 eV·e^–1^ vs 10.2 eV·e^–1^ using the revised scale of electronegativity.^75^

b The solid electrolyte inductive effect model of Krauskopf et al.^35^ borrows from the general concept of an inductive effect, which has been used to explain trends in a number of properties within classes of functional materials.^42,76^
