# Supplementary material for: Evidence for a Solid-Electrolyte Inductive Effect in the Superionic Conductor Li10Ge1–xSnxP2S12
Source: J Am Chem Soc. 2020 Dec 7;142(50):21210–9. doi: 10.1021/jacs.0c10735 (PMC8016198; doi:10.1021/jacs.0c10735)
Supplement: Supplementary file 1 — ja0c10735_si_001.pdf [file ja0c10735_si_001.pdf]

Evidence for a Solid-Electrolyte Inductive Effect in the Superionic Conductor $Li_{10}Ge_{1-x}Sn_xP_2S_{12}$

Sean P. Culver[a,b,$], Alexander G. Squires[c,d,$], Nicolò Minafra[e], Callum W. F. Armstrong[c], Thorben Krauskopf[a,b], Felix Böcher[a,b], Cheng Li[f], Benjamin J. Morgan*[c,d], Wolfgang G. Zeier*[,e]

[a]*Institute of Physical Chemistry, Justus-Liebig-University Giessen, Heinrich-Buff-Ring 17, D-35392 Giessen, Germany.*

[b]*Center for Materials Research (LaMa), Justus-Liebig-University Giessen, Heinrich-Buff-Ring 16, D-35392 Giessen, Germany.*

[c]*Department of Chemistry, University of Bath, Claverton Down, UK*

[d]*The Faraday Institution, Didcot OX11 0RA, U.K.*

[e]*Institute of Inorganic and Analytical Chemistry, University of Münster, Correnstrasse 30, 48149 Münster, Germany*

[f]*Jülich Centre for Neutron Science JCNS, Forschungszentrum Jülich GmbH, Outstation at SNS, 1 Bethel Valley Road, Oak Ridge, Tennessee 37831-6473, United States.*

**B.J.Morgan@bath.ac.uk; wzeier@uni-muenster.de*

[$]These authors contributed equally to the manuscript

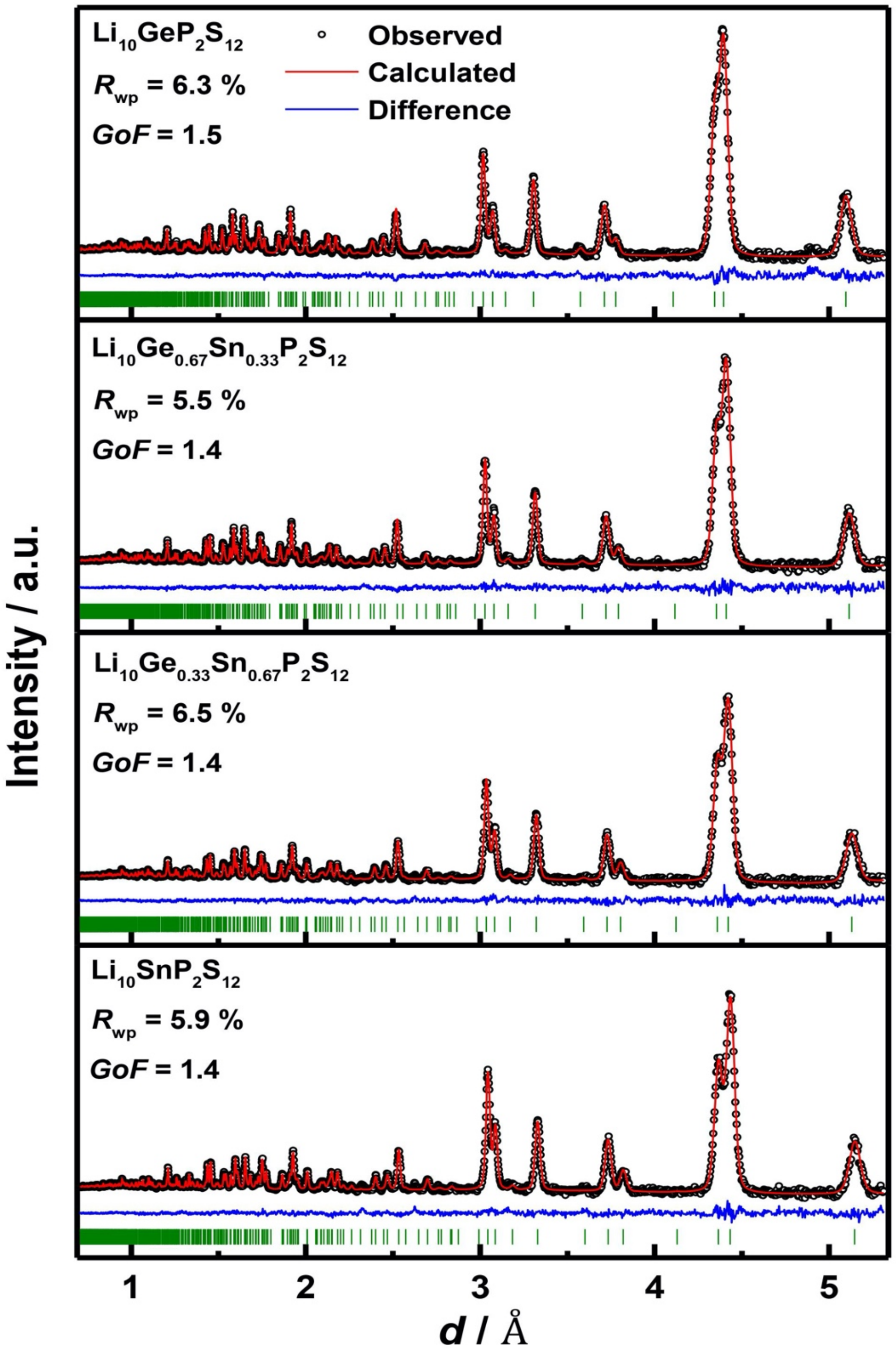

Figure S1: Representative Rietveld refinements of neutron diffraction data at 300 K for $Li_{10}Ge_{1-x}Sn_xP_2S_{12}$. R_{wp} and GoF are the weighted profile residuals and goodness-of-fit, respectively.

Table S1: Constraints used to refine the $Li_{10}Ge_{1-x}Sn_xP_2S_{12}$ structures from powder time-of-flight neutron diffraction data.

Atom	Wyckoff Site	x/*a*	y/*b*	z/*c*	Occ.	B_{eq} / Å^2
Li1	16*h*	Pos1	Pos2	Pos3	[5-Occ1-(4*Occ2)-Occ3]/4	Var1
Li2	4*d*	0.25	0.25	Pos4	Occ1	Var2
Li3	16*h*	Pos5	Pos6	Pos7	Occ2	Var3
Li4	4*c*	0.25	0.75	Pos8	Occ3	Var4
Ge/Sn1	4*d*	0.25	0.25	Pos9	0.5-Occ4	Var5
Ge/Sn2	4*d*	0.25	0.25	Pos9	Occ4	Var5
P1	4*d*	*0.25*	0.25	Pos9	0.5	Var5
P2	2*b*	0.25	0.75	0.75	1.0	Var6
S1	8*g*	0.25	Pos10	Pos11	1.0	Var7
S2	8*g*	0.25	Pos12	Pos13	1.0	Var8
S3	8*g*	0.25	Pos14	Pos15	1.0	Var9

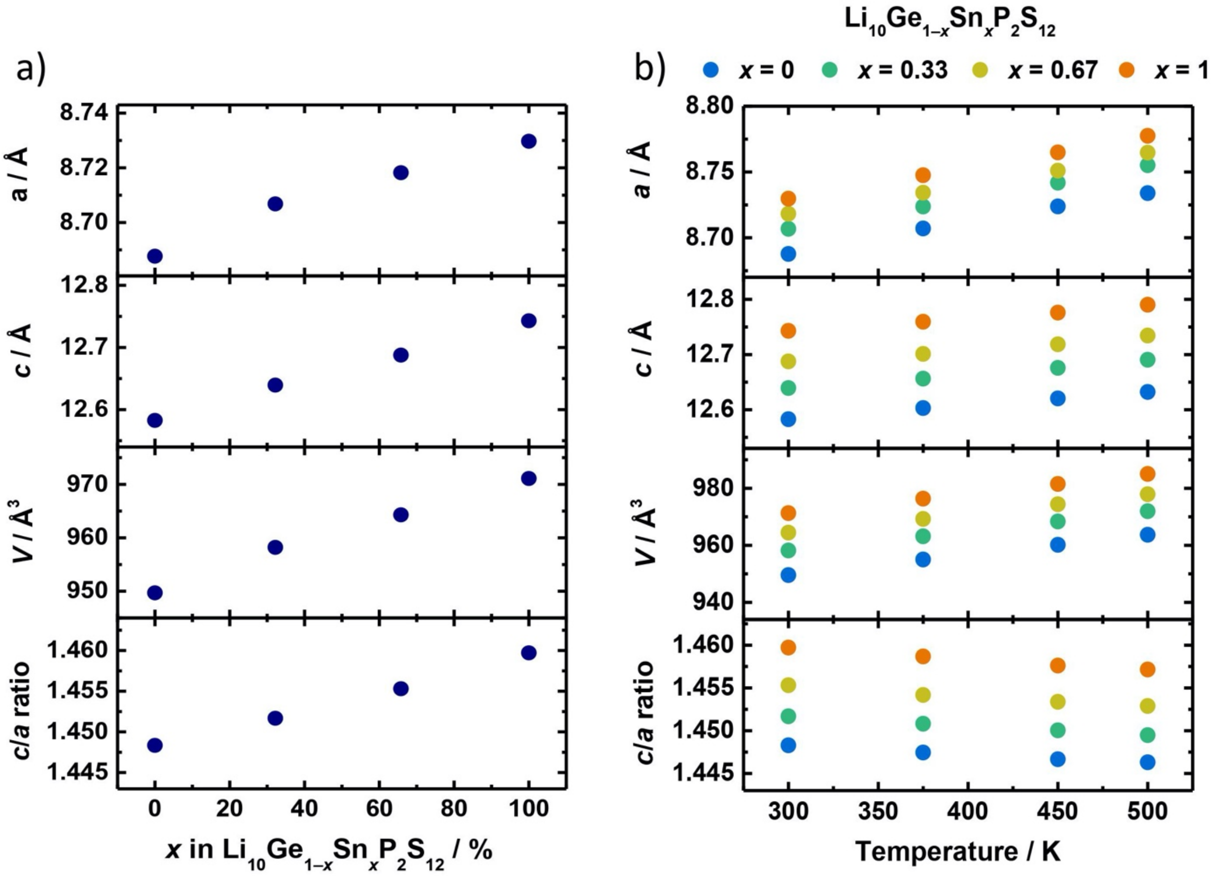

Figure S2: (a) Dependence of the room-temperature lattice parameters, lattice volume and c/a ratio in $Li_{10}Ge_{1-x}Sn_xP_2S_{12}$ on the refined Sn occupancies. (b) Temperature dependence of the lattice parameters, lattice volume and c/a ratio in $Li_{10}Ge_{1-x}Sn_xP_2S_{12}$.

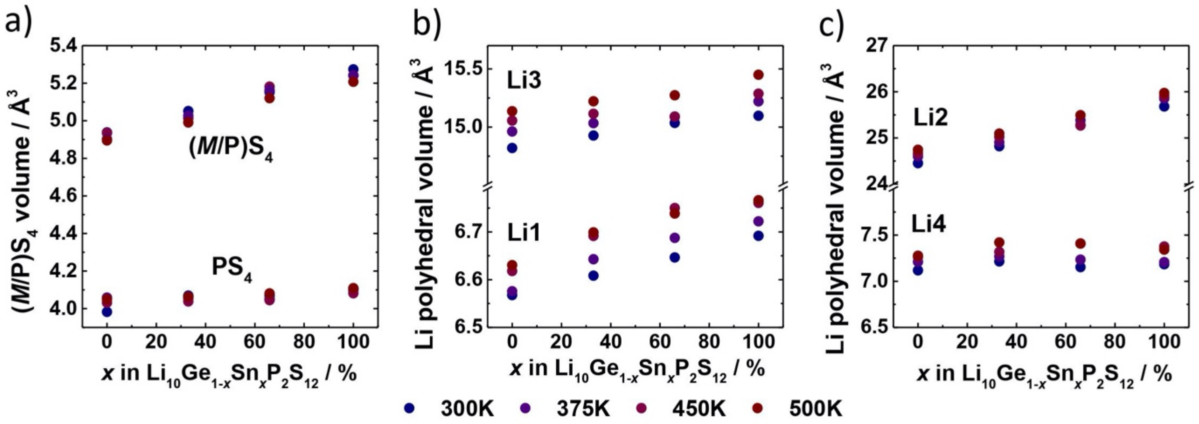

Figure S3: Effect of temperature and composition on (a) PS_4, $(M/P)S_4$ and (b,c) lithium polyhedral volumes.

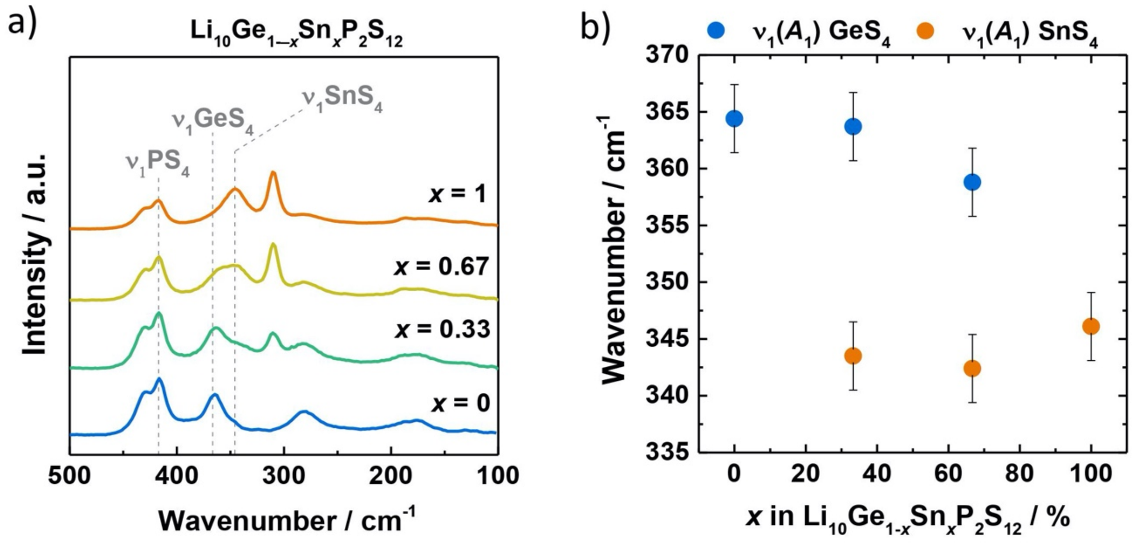

Figure S4: (a) Raman spectra with the relevant tetrahedral vibrations assigned. An additional Raman band at 310 cm^{-1} corresponds to 2-4 wt% Li_2SnS_3 side phase. (b) Raman shifts for the symmetric stretching modes of the GeS_4 and SnS_4 tetrahedra.

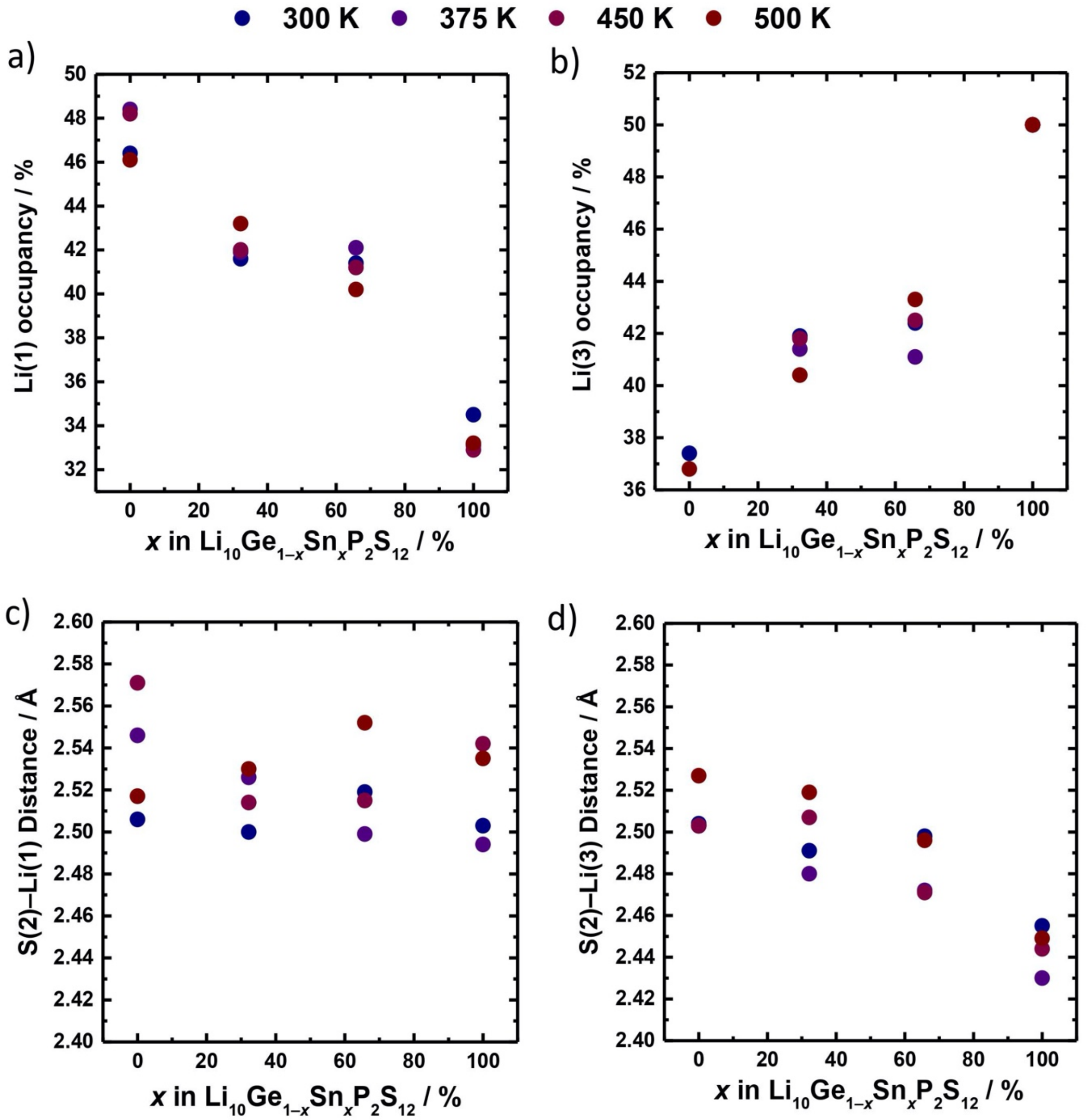

Figure S5: All data obtained from Rietveld refinements at each temperature relative to lithium occupancies (a, b) and S – Li distances (c, d)

Table S2: Room temperature ionic conductivity (σ_{RT}) and activation energy (E_A) of $Li_{10}Ge_{1-x}Sn_xP_2S_{12}$ from reference 35.

Composition	E_A / eV	σ_{RT} / mScm^{-1}
$Li_{10}GeP_2S_{12}$	0.274(3)	7.6(3)
$Li_{10}Ge_{0.66}Sn_{0.33}P_2S_{12}$	0.275(3)	6.4(3)
$Li_{10}Ge_{0.33}Sn_{0.66}P_2S_{12}$	0.285(3)	4.8(2)
$Li_{10}SnP_2S_{12}$	0.304(3)	3.8(2)
